# Nutritional and Functional Impact of Acute SARS-CoV-2 Infection in Hospitalized Patients

**DOI:** 10.3390/jcm11092424

**Published:** 2022-04-26

**Authors:** Angie Gómez-Uranga, Javier Guzmán-Martínez, Pedro Jesús Esteve-Atiénzar, Philip Wikman-Jorgensen, Juan Manuel Núñez-Cruz, Leticia Espinosa-del-Barrio, Isidro Hernández-Isasi, Francisco J. Pomares-Gómez, Eva Perelló-Camacho, Nuria Fernández-García, Ángel Sánchez-Miralles, Vicente Giner-Galvañ

**Affiliations:** 1 Internal Medicine Service, University Hospital San Juan de Alicante, 03550 Alicante, Spain; guzmanjavier1994@gmail.com (J.G.-M.); wikman_phi@gva.es (P.W.-J.); nunezcruz.juanmanuel21@gmail.com (J.M.N.-C.); leticiaespdelbarrio@gmail.com (L.E.-d.-B.); isidro.hrdz94@gmail.com (I.H.-I.); ginervicgal@gmail.com (V.G.-G.); 2 Foundation for the Promotion of Health and Biomedical Research of Valencia Region (FISABIO), 46020 Valencia, Spain; pomares_fra@gva.es (F.J.P.-G.); evapc89@hotmail.com (E.P.-C.); n.fernandez@umh.es (N.F.-G.); an.sanchezm1@coma.es (Á.S.-M.); 3 Endocrinology and Nutrition Section, University Hospital San Juan de Alicante, 03550 Alicante, Spain; 4 Rehabilitation Service, University Hospital San Juan de Alicante, 03550 Alicante, Spain; 5 Intensive Care Unit, University Hospital San Juan de Alicante, 03550 Alicante, Spain; 6 Clinical Medicine Department, University Miguel Hernández de Elche, 03550 Alicante, Spain

**Keywords:** SARS-CoV-2, malnutrition, sarcopenia, frailty

## Abstract

Aim: To assess the prevalence of malnutrition, frailty, and sarcopenia and the relationships between them in patients hospitalized for COVID-19. Methods: This was a cross-sectional study of the prevalence, determinants, and associations between malnutrition (GLIM 2019 criteria), sarcopenia (SARC-F scale, dynamometry, and calf circumference), and frailty (FRAIL scale) upon discharge following hospitalization for COVID 19. Results: A total of 101 patients (67.3% men, mean age 66.3 years) were recruited. Malnutrition was diagnosed in 49.5%, sarcopenia in 32.7%, and frailty in 28.7% of patients. Of the patients with malnutrition, 48% were also sarcopenic, and 42% were frail. There was a significant association between malnutrition and the severity of pneumonia according to the CURB-65 scale (odds ratio [OR] 2.61, *p* = 0.036), between sarcopenia and a Barthel score lower than 60 points (OR 29.52, *p* < 0.001), and between frailty and both a Barthel score lower than 60 points (OR 32.27, *p* < 0.001) and a length of hospital stay of over 30 days (OR 9.11, *p* = 0.008). Conclusions: Malnutrition, sarcopenia, and frailty are prevalent and interrelated entities in patients hospitalized for acute SARS CoV-2 infection, especially in patients with greater baseline functional impairment prior to admission and a higher infection severity.

## 1. Introduction

Hospitalization has well-known negative effects on nutritional and functional status. In the case of admission to the intensive care unit (ICU) [1], these effects are accentuated by the catabolic changes produced by inflammation [2], the diagnostic–therapeutic procedures associated with hospitalization, and the admission itself. These effects have been studied in patients with viral pneumonia, such as influenza [3], and in related animal models [4]. Similar to the flu, SARS-CoV-2 disease induces a hypercatabolic state due to inflammatory hyperactivity, the final expression of which leads to acute respiratory distress syndrome (ARDS) in up to 5% of patients. ARDS is, in turn, associated with prolonged ICU stays and high mortality [5,6,7]. Recent studies have described a prevalence of malnutrition of about 38.5% to 50% or 50% to 70% in cases of severe COVID-19 [8,9], and they have explored how its presence affects mortality [10,11]. Older people are especially susceptible to the development of malnutrition and sarcopenia under these circumstances [12,13], while symptoms such as sensory deficit (anosmia and dysgeusia) and gastrointestinal disturbances are other risk factors for malnutrition and sarcopenia that are specific to COVID-19 [14]. 

Another factor closely related to malnutrition and sarcopenia is frailty, understood as a state of greater vulnerability to stressors arising from diminished or dysregulated physiological reserves [15]. This factor has also been linked to an increased risk of severe COVID-19 [16] and in-hospital mortality [17].

In summary, malnutrition, sarcopenia, and frailty seem to develop frequently during admissions for SARS-CoV-2, interacting with disease severity and age to cause important impacts on patients’ functional and vital prognoses. The variety of criteria used for assessing these conditions and the heterogeneity of the populations considered limit the generalizability of research results. Moreover, although these conditions are closely related, no studies have been published that examine these three conditions simultaneously. Thus, the aim of this study was to assess the prevalence of malnutrition, sarcopenia, and frailty in adults infected with COVID-19 and to explore the interrelationships among them and the associated factors. 

## 2. Materials and Methods

### 2.1. Study Design 

This was a cross-sectional descriptive study analyzing the nutritional and functional status at discharge in patients admitted for COVID-19 at the University Hospital San Juan de Alicante from 29 April 2020 to 28 February 2021.

### 2.2. Participants 

Eligible patients were aged 18 years or older and were admitted to and discharged from the hospital during the study period because of acute SARS-CoV-2 infection, confirmed microbiologically by PCR. Patients in whom SARS-CoV-2 infection was not confirmed microbiologically, those admitted for something other than COVID-19 infection (regardless of PCR positivity), pregnant women, and patients who died during admission were excluded. All participants signed informed consent. Recruitment was carried out by convenience sampling, and the general characteristics of our sample were similar to those of the admitted patients for COVID-19 in our center.

### 2.3. Study Variables 

Variables collected on admission included demographic (age and sex) and clinical variables (comorbidities and pneumonia severity) as well as overall functional status, with a score of 60 points or less on the Barthel index indicating dependence [18]. The severity of pneumonia was assessed using the FINE-PSI [19] and CURB-65 [20] scales; patients meeting the criteria for FINE-PSI class IV or above and with a CURB-65 score of more than 2 points were classified as having severe pneumonia. The rest of the variables were collected on discharge. Nutritional status was assessed following the criteria of the Global Leadership Initiative on Malnutrition (GLIM) [21], which defines a patient with malnutrition as a patient with one etiological criterion, in this case, acute SARS-CoV-2 infection, and at least one of the following phenotypic criterion: weight loss of >5% in the previous 6 months, low BMI (<20 kg/m^2^), or low muscle mass. Muscle mass was estimated on the basis of calf circumference (cm) and muscle strength (kg) using the dominant arm strength test with a JAMAR dynamometer [22]. Low muscle mass was defined as a calf circumference of less than 31 cm [23]. Decreased muscle strength was defined as a dynamometer value lower than the normal value according to sex and age group [22]. Patients were considered to have sarcopenia if they had a score of greater than 4 points on the SARC-F scale [24], while frailty was defined as a score of 3 or more on the FRAIL scale [25]. The general feeding regime during hospitalization was around 1800–2000 kcal per day, about 15–20% of which came from protein. Intubated patients received enteral nutrition with a high-protein, hypercaloric formula.

### 2.4. Statistical Analysis 

The SPSS 15 program (IBM) was used for the statistical analysis. A descriptive analysis was performed to obtain prevalence estimates for categorical variables. Quantitative variables following a normal distribution are expressed as means (standard deviation, SD), whereas non-parametric variables are described as medians (interquartile range, IQR). Categorical variables were compared using the chi-squared statistic, and quantitative variables were compared using Student’s t-test, while the ANOVA test was used to test associations between categorical variables and a quantitative variable. Non-parametric quantitative variables were compared with dichotomous variables using the Mann–Whitney U test, and they were compared with multicategorical variables using the Kruskal–Wallis test. A *p*-value of less than 0.05 was considered significant. Variables with clinical relevance and statistical associations yielding a *p*-value of less than 0.10 in stepwise regression were selected for inclusion in a multivariable binary logistic regression model. Results were expressed as adjusted odds ratios (ORs) with 95% confidence intervals (CIs).

## 3. Results

### 3.1. Participant Characteristics 

The study population comprised 101 patients, of whom 67.3% (*n* = 68) were men, with a mean age of 66.3 (95% confidence interval (CI) 50.8–81.8) years. The most frequent comorbidities were hypertension (58.4%), dyslipidemia (35.6%), and obesity, defined as a body mass index (BMI) of at least 30 kg/m^2^ (30.7%). Table 1 presents the rest of the comorbidities present on admission. Regarding patients’ autonomy for performing the basic activities of daily living as measured by the Barthel scale, 79.2% of the patients were considered independent, and 20.8% were severely or totally dependent.

The median interval from symptom onset to admission was 7 days (IQR 4–13), while the median length of hospital stay was 16 days (IQR 8–26.5); 17.2% of the participants were admitted for longer than one month.

At the time of admission, 28.7% of patients had severe pneumonia according to the FINE-PSI scale and 35.1% had severe pneumonia according to the CURB-65 scale, while 38.6% of patients met the criteria for ARDS. In 24.8% of the cases, admission to the ICU was required during hospitalization. Among these patients, 84% were men, the mean age was 60.4 years (95% CI: 52.3–68.5), the median length of ICU stay was 17 days (IQR 7–32.5), and the median length of hospital stay was 33 days (IQR 23.5–54). The patients who did not require intensive care were mostly men (61.8%), with a mean age of 68.2 years (95% CI 51.5–84.9) and a median hospital stay of 12 days (IQR 7–17.7). Table 1 provides further details on patient characteristics.

### 3.2. Prevalence of Malnutrition, Sarcopenia and Frailty

**Figure 1:**
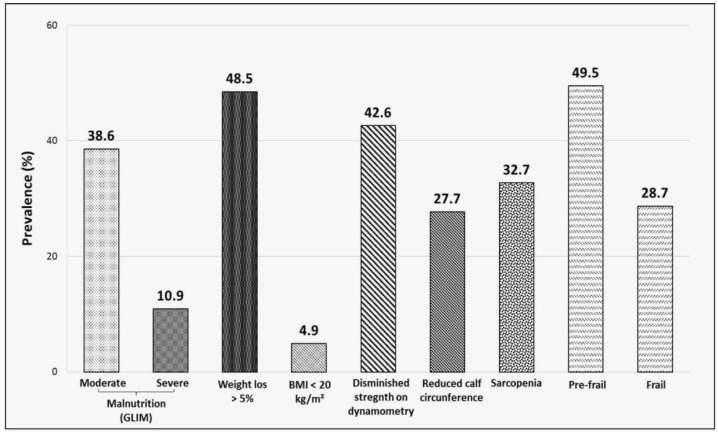
Prevalence of malnutrition and its components, sarcopenia, and frailty in the study sample. Prevalence expressed as percentage (%). Diagnosis of malnutrition was according to GLIM 2019 criteria (Global Leadership Initiative of Malnutrition). Sarcopenia was diagnosed according to the SARC-F screening tool. Frailty categorization by FRAIL scale (Fatigue, Resistance, Ambulation, Illnesses, Loss of Weight). BMI: body mass index.

About half (49.5%) of the participants met the GLIM criteria for malnutrition, with 38.6% defined as moderate and 10.9% defined as severe. The most prevalent phenotypic criteria for malnutrition were weight loss of >5% (48.5%); reduced muscle strength, as defined by dynamometry (42.6%); reduced calf circumference (27.7%); and BMI < 20 kg/m^2^ (4.9%). The prevalence of sarcopenia, as estimated using the SARC-F questionnaire, was somewhat lower at 32.7%, while 28.7% of the sample was classified as frail and 49.5% as pre-frail according to the FRAIL test (Figure 1).

An analysis of the interrelationships between malnutrition, sarcopenia, and frailty showed that among patients with malnutrition, 48% also met the criteria for sarcopenia, and 42% met the criteria for frailty (Table 2). Of those diagnosed with sarcopenia, 72.7% had malnutrition, and 69.7% had frailty. Finally, 72.4% of patients with frailty also had malnutrition, and 79.3% had sarcopenia.

### 3.3. Determinants of Malnutrition, Sarcopenia and Frailty

Table 1 includes the results of the bivariable analysis of factors associated with malnutrition, sarcopenia, and frailty, and Table 3 shows the adjusted ORs and 95% CIs from the multivariable analysis.

The prevalence of malnutrition showed significant differences (*p* = 0.014) according to age groups, with higher prevalence in patients aged 60 and 74 years (65.7%, *n* = 21) compared with younger (31.6%, *n* = 12) and older participants (54.8%, *n* = 17). Statistically significant differences were also observed between patients who were admitted versus those who were not admitted to the ICU (72% vs. 42.1%; *p* = 0.01), those with a length of stay over versus under 30 days (83.3% vs. 42.2%; *p* < 0.01), and in participants with more versus less severe pneumonia (FINE-PSI: class V 83.3% vs. class I 27.3%; *p* = 0.042; CURB-65: grade 3 75.0% vs. grade 0 24.1%; *p* = 0.002). There was also more malnutrition in dependent versus autonomous patients (Barthel ≤ 60 points 66.7% vs. >60 points 45.0%; *p* = 0.077) and in those who had ARDS versus those who did not have ARDS on admission (61.5% vs. 41.9%, *p* = 0.055); however, these differences were not statistically significant.

With regard to sarcopenia, we similarly observed significant differences according to age (*p* < 0.001), with patients aged 75 years or more showing a much higher prevalence (61.3%) compared with those under 60 (15.8%) and those 61 to 74 years old (25.0%). The differences were also statistically significant in participants with a Barthel index of 60 points or less versus those with a score of more than 60 (85.7% vs. 18.7%; *p* < 0.001), and again according to the severity of pneumonia (FINE-PSI: class I 0% vs. V 50%, *p* = 0.004 and CURB-65: grade 0 10.3% vs. grade 3 66.7%, *p*= 0.001). No statistically significant differences were observed in the case of admission to the ICU, prolonged length of stay, presence of ARDS on admission, or in terms of the comorbidities studied. 

Finally, frailty was significantly associated with age (prevalence 58.1% in those aged ≥ 75 years versus 21.9% in patients aged 61–74 and 10.5% in those <60 years; *p* < 0.001); dependence (Barthel ≤ 60 81.0% vs. Barthel > 60 15.0%; *p* < 0.001); prolonged length of hospital stay (≥30 days 50% vs. <30 days 24.1%; *p* = 0.028); and severe pneumonia (FINE-PSI *p* = 0.010; CURB-65 *p* = 0.001). The comorbidities that were significantly associated with frailty were hypertension (*p* = 0.024), diabetes mellitus (*p* = 0.004), chronic kidney disease (*p* = 0.021), and chronic heart failure (*p* = 0.021).

In the multivariable analysis, the association between malnutrition and the severity of pneumonia as determined by CURB-65 remained significant (OR 2.61, *p*= 0.036). Both sarcopenia and frailty were significantly associated with a Barthel index under 60 points at admission (OR 29.52, *p* < 0.001 and OR 32.27, *p* = 0.001, respectively). Frailty was also associated with a stay of more than 30 days (OR 9.11, *p* = 0.008). Obesity showed a protective effect against sarcopenia (OR 0.20; *p* = 0.036).

## 4. Discussion

In our study population of patients admitted for COVID-19, the prevalence of malnutrition at discharge was 49.5%; the prevalence of sarcopenia was 32.7%; and the prevalence of frailty was 28.7%. These entities were shown to be strongly interrelated. Moreover, malnutrition was associated with severe pneumonia, as measured with the CURB-65 scale, while baseline dependency (Barthel score of ≤60 points) was related to both sarcopenia and frailty. Frailty was also related to a length of stay of more than 30 days. Obesity was associated with less sarcopenia.

Although in general, older people have a higher risk of developing malnutrition after an acute condition, the mean age in our study was just 66.3 years (SD 15.5), and the highest prevalence of malnutrition was in the group aged 60 to 74 years, with no evidence of an independent association between age and malnutrition. Thus, during hospital admission for SARS-CoV-2, the presence and impact of this entity must be considered in relatively younger populations, such as those aged 60 years. Other similar studies using the GLIM criteria have reported findings consistent with ours. Bedock et al. [26] reported a prevalence of malnutrition of 42.1% (moderate 23.7%, severe 18.4%) in 114 French patients with a mean age of 55.9 years (standard deviation [SD] 15.9). Rouget et al. [27] described a somewhat lower prevalence (37.5%) in a sample of 80 French patients with a median age of 59.5 (IQR 49.5–68.5) years. On the other hand, in 355 Filipinos (mean age 54.7, SD 15.2 years), Larrazabal et al. observed a prevalence reaching 71.8%. Unlike other authors and the authors of the current study, these researchers analyzed the prevalence of malnutrition using the modified global subjective assessment method [28]. The use of different diagnostic criteria, as well as the marked population differences, could explain the differences in the observed prevalence. In any case, results from the previous studies, carried out in predominantly white populations and with the GLIM criteria, are in keeping with ours, suggesting that about half the patients hospitalized for COVID-19 present malnutrition, regardless of age.

In agreement with the literature, we observed that the severity of COVID-19 infection on admission was related to the development of malnutrition: the higher the score on the FINE and CURB-65 scales, the greater the prevalence of malnutrition. The same trend is apparent in other studies, including Bedock et al.’s [26], where the prevalence of malnutrition was 66.7% in patients admitted to the ICU compared with 37.5% in those who were not. Although we did not assess the prevalence on admission, this pattern was also reproduced in our study; 72% of those who were admitted to the ICU, versus 42.1% of those who were not, presented malnutrition, although this difference was not significant in the multivariate analysis. For their part, Allard et al. [9] did not observe an association between severe COVID-19 and malnutrition, defined as low BMI or weight loss greater than 5%; however, greater disease severity was associated with the risk of developing malnutrition. On the basis of the literature and our own findings, severe COVID-19 appears to be a risk factor for malnutrition, and the differences across reports can be explained by the heterogeneous criteria used to establish severity. The use of baseline risk scales could be of interest insofar as they would help identify a subpopulation that would benefit from early malnutrition prevention measures. Indeed, the relationship with disease severity points to a direct role of the infection in the development of malnutrition, over and above the general factors related to hospitalization.

Unlike previous studies, we evaluated muscle both quantitatively (muscle mass) and from a functional perspective (muscular strength), with all results indicating the negative impact of the infection on the muscle. In this sense, almost half (42.6%) of the patients evaluated showed a decrease in muscle strength in the dominant arm, and over a quarter (27.7%) presented a loss of muscle mass. These proportions were significant, and the differences between them can be explained by the fact that it takes more time to lose muscle mass than strength in situations of inactivity and hypercatabolism [29]. As already observed for malnutrition, there was a significantly higher prevalence of sarcopenia in the most severe cases, again pointing to the direct effect of the infection, as previously indicated in the literature.

In fact, the few studies similar to ours have reported a higher prevalence of sarcopenia than we reported. Wierdsma et al. [30], who also assessed sarcopenia by means of the SARC-F questionnaire, observed almost twice the prevalence of sarcopenia (73%) in a sample of 407 patients from four different hospitals, with a mean age similar to ours (64.8 years SD 12.4). The reasons for this difference may reside in the higher percentage of patients who required ICU admission (60.2%) compared with our participants (24.8%) [2,4]. We also observed a significantly higher prevalence of sarcopenia in those over 75 years of age (61.3%, *p* < 0.001). Riesgo et al., also in Spain, observed a similar relationship using the same questionnaire in a population with a mean age of 86.1 years, finding a prevalence of sarcopenia of 80.2% [13]. This proportion is also considerably higher than that found in our study, probably because of the combined impact of primary sarcopenia (attributable to age-related muscle loss) and sarcopenia secondary to acute inflammation. The results of our multivariable analysis support that hypothesis, showing a significant relationship between sarcopenia and a pre-admission Barthel index of less than 60 as a probable expression of worse baseline muscle function due to disuse [31]. On the other hand, no differences were found in the patients admitted to the ICU or with a stay of more than 30 days. However, once these patients were transferred to a conventional hospitalization ward or flagged as having a prolonged stay, rehabilitation exercises and nutritional support were started early, which would have mitigated the negative impact on muscle.

The protective effect of obesity against sarcopenia is striking. This result is probably due in part to selection bias, as this comorbidity is a risk factor associated with mortality from COVID-19 [32], and our population was made up of people who survived it. It is possible that the energy reserve effect predominates over the restrictive ventilatory compromise in obese patients who survive, who could then preserve muscle mass as an energy source. In fact, some authors have described a positive association between BMI and muscle mass, probably because heavier people bear more weight and consequently perform more muscle work; they may also have a higher protein intake, which in turn produces a greater anabolic effect [33].

Regarding frailty, Ma et al. used the FRAIL scale and obtained a prevalence of 31.6% in 114 participants with a median age of 67 years [17], while Aw et al. applied the Rockwood Clinical Frailty Scale and observed a prevalence of 54.4% [18]. These studies, unlike ours, assessed patients upon admission, associating baseline frailty with higher mortality. In any case, both our study and Ma et al.’s found a similar prevalence using the FRAIL scale, while Aw et al.’s estimate was higher than both using a different definition. Regarding the factors associated with the development of frailty, we identified several comorbidities, which was expected since the FRAIL scale contains an item on the presence of comorbidities. The severity of pneumonia according to both scales, a Barthel score of less than 60, and a stay of more than 30 days were other factors associated with frailty.

Malnutrition, sarcopenia, and frailty are not isolated conditions, and they frequently coexisted in our participants. Not all patients presenting diminished strength by dynamometry or reduced calf circumference met the 4-point cutoff for sarcopenia on the SARC-F questionnaire, suggesting that assessing sarcopenia requires the measurement of strength parameters, not just the completion of validated questionnaires. In that regard, an assessment with dynamometry may be the best option because of the direct relationship between strength and function.

The present study has several limitations, the most important of which are inherent to its cross-sectional design. Sarcopenia, malnutrition, and frailty may have been present prior to hospitalization in some patients, especially in those with moderate or severe dependence. Another limitation is the small sample size, which—though similar to other studies—limits the statistical power of some associations. Undoubtedly, the greatest limitation is that the assessments were carried out close to the end of admission, precluding a comparison of outcomes at admission and discharge. However, the disease severity in many patients and the fact that COVID-19 is a highly contagious infection limit the feasibility of this approach. One limitation that this study shares with previous ones, and which we hope to mitigate in future studies, is the lack of assessment of associations between malnutrition/sarcopenia/frailty and clinical outcome variables.

On the other hand, the main strength of the study is that nutritional and functional aspects were analyzed in the context of routine clinical practice, allowing the findings to be extrapolated to future clinical actions to detect and tackle these entities in patients hospitalized for SARS-CoV-2. Moreover, the use of validated scales that are applicable in clinical practice undoubtedly gives greater consistency to the results obtained. We also analyzed hospitalizations with different levels of severity, not only critical patients. Finally, to our knowledge, this is the first study to assess the three interrelated conditions of malnutrition, sarcopenia, and frailty in the same sample.

## 5. Conclusions

In conclusion, hospitalization for acute SARS CoV-2 infection has a negative impact on nutritional and functional status, especially when the clinical presentation of this disease is more severe and in patients with a poorer basal functional status. These results suggest that the influence depends directly on the viral infection rather than general aspects related to admission. Despite the current absence of evidence on the impact on health outcomes, the high prevalence of malnutrition in patients hospitalized for COVID-19 points to the need for nutritional screening, follow-up, and intervention when necessary, especially in more severe cases. 

## Figures and Tables

**Table 1:** Patient characteristics according to prevalence of malnutrition, sarcopenia, and frailty.

Variable	Total (*n* = 101) *n* (% col)	Malnutrition (*n* = 50) *n* (% row)	p *	Sarcopenia (*n* = 33) *n* (% row)	p *	Frailty (*n* = 29) *n* (% row)	p *
Age in years, mean 66.3 (95% CI 50.8–81.8)							
≤60 years	38 (37.6)	12 (31.6)	0.014	6 (15.8)	<0.001	4 (10.5)	<0.001
61–74 years	32 (31.7)	21 (65.7)		8 (25.0)		7 (21.9)	
≥75 years	31 (30.7)	17 (54.8)		19 (61.3)		18 (58.1)	
Sex							
Male	68 (67.3)	35 (51.5)	0.57	20 (29.4)	0.32	17 (25)	0.236
Female	33 (32.7)	15 (45.5)		13 (39.4)		12 (36.4)	
Comorbidities							
Hypertension	59 (58.4)	27 (45.8)	0.37	23 (39)	0.11	22 (37.3)	0.024
Dyslipidemia	35 (35.6)	20 (55.6)	0.37	15 (41.7)	0.15	13 (36.1)	0.22
Obesity	31 (30.7)	11 (35.5)	0.061	6 (19.4)	0.058	9 (36.1)	0.96
Diabetes mellitus	20 (19.8)	12 (60)	0.30	10 (50)	0.065	11 (55)	0.004
COPD	15 (14.9)	9 (60)	0.38	6 (40)	0.51	7 (46.7)	0.096
Chronic kidney disease	10 (9.9)	6 (60)	0.48	6 (60)	0.052	6 (60)	0.021
Heart failure	10 (9.9)	7 (70)	0.17	6 (60)	0.052	6 (60)	0.021
Asthma	6 (5.9)	3 (50)	0.98	1 (16.7)	0.39	1 (16.7)	0.30
Immunocompromised	4 (3.9)	1 (25)	0.32	0	0.16	0	0.20
Liver disease	3 (2.9)	1 (33)	0.57	1 (33)	0.98	1 (33)	0.86
HIV	2 (1.9)	0	0.37	0	0.28	1 (50)	0.24
Barthel index, median 100 points (IQR 87.5–100)							
≤60 points (dependent)	21 (20.8)	14 (66.7)	0.077	18 (85.7)	<0.001	17 (81.0)	<0.001
>60 points (autonomous)	80 (79.2)	36 (45.0)		15 (18.7)		12 (15.0)	
Length of hospital stay, median 16 days (IQR 8–26.5)							
<30 days	83 (82.2)	35 (42.2)	0.002	25 (30.1)	0.12	20 (24.1)	0.028
≥30 days	18 (17.2)	15 (83.3)		8 (44.4)		9 (50)	
Admission to ICU							
Yes	25 (24.8)	18 (72.0)	0.010	8 (32.0)	0.93	7 (28.0)	0.33
No	76 (75.2)	32 (42.1)		25 (32.9)		22 (28.9)	
FINE-Pneumonia Severity Index † on admission							
I	11 (10.9)	3 (27.3)	0.042	0 (0)	0.004	0 (0)	0.010
II	32 (31.7)	13 (40.6)		6 (18.8)		5 (15.6)	
III	29 (28.7)	13 (44.8)		11 (37.9)		8 (27.6)	
IV	23 (22.8)	16 (69.6)		13 (56.5)		13 (56.5)	
V	6 (5.9)	5 (83.3)		3 (50.0)		3 (50.0)	
CURB-65 ‡ pneumonia severity score on admission (points)							
0	29 (28.7)	7 (24.1)	0.002	3 (10.3)	0.001	2 (6.9)	0.001
1	36 (35.6)	17 (47.2)		10 (27.8)		9 (25.0)	
2	28 (27.2)	20 (71.4)		14 50.0)		13 (46.4)	
3	8 (7.9)	6 (75.0)		6 (66.7)		5 (62.5)	
ARDS on admission							
Yes	39 (38.6)	24 (61.5)	0.055	10 (25.6)	0.196	11 (28.2)	0.59
No	62 (61.4)	26 (41.9)		23 (37.1)		18 (29.5)	

Diagnosis of malnutrition was according to GLIM (Global Leadership Initiative of Malnutrition) 2019 criteria. Sarcopenia was diagnosed according to the SARC-F screening tool. Frailty categorization by FRAIL scale (Fatigue, Resistance, Ambulation, Illnesses, Loss of Weight). In the columns “Malnutrition”, “Sarcopenia”, and “Frailty”, the percentage corresponds to the frequency of that entity with every given variable. ARDS: acute respiratory distress syndrome; CI: confidence interval; COPD: chronic obstructive pulmonary disease; ICU: intensive care unit; IQR: interquartile range; * Bivariate analysis; † Classes IV and V = severe pneumonia. ‡ ≥ 2 points indicates severe pneumonia.

**Table 2:** Relationships between malnutrition, sarcopenia, and frailty.

	Malnutrition GLIM (*n* = 50) *n* (%)	Sarcopenia SARC-F (*n* = 33) *n* (%)	Frailty FRAIL (*n* = 29) *n* (%)
Malnutrition GLIM		24 (72.7)	21 (72.4)
Moderate	-	17 (51.5)	13 (44.8)
Severe	-	7 (21.2)	8 (27.6)
Weight loss > 5%	-	18 (54.5)	14 (48.3)
BMI < 20 kg/m^2^	-	3 (9.1)	2 (6.9)
Sarcopenia			
SARC-F	24 (48.0)	-	23 (79.3)
Diminished strength on dynamometry	26 (52.0)	27 (81.8)	24 (82.6)
Reduced calf circumference	26 (52.0)	20 (60.6)	19 (65.5)
Frailty (FRAIL)			
Pre-frail	26 (52.0)	10 (30.3)	-
Frail	21 (42.0)	23 (69.7)	-

Diagnosis of malnutrition was according to GLIM 2019 criteria (Global Leadership Initiative of Malnutrition). Sarcopenia was diagnosed according to the SARC-F screening tool. Frailty categorization by FRAIL scale (Fatigue, Resistance, Ambulation, Illnesses, Loss of Weight). BMI: body mass index. Prevalence expressed as percentage (*n* = 101).

**Table 3:** Results of multivariable analysis of associations between explanatory variables and the development of malnutrition, sarcopenia, and frailty in patients admitted for COVID-19 infection (*n* = 101).

Variable	Malnutrition		Sarcopenia			Frailty
	OR (95% CI)	*p*	OR (95% CI)	*p*	OR (95% CI)	*p*
Age	1.25 (0.52–3.03)	0.62	1.21 (0.48–3.06)	0.63	2.37 (0.76–7.36)	0.17
Sex	*		*		*	
Hypertension	*		*		1.74 (0.30–9.97)	0.53
Dyslipidemia	*		*		*	
Obesity	0.38 (0.13–1.06)	0.064	0.20 (0.04–0.90)	0.036	*	
Diabetes	*		1.65 (0.34–8.09)	0.54	1.30 (0.24–7.05)	0.76
COPD	*		*		0.34 (0.06–1.88)	0.22
Chronic kidney disease	*		*		0.19 (0.01–2.73)	0.22
Heart failure	*		2.82 (0.40–20.00)	0.30	1.53 (0.11–22.29)	0.76
Asthma	*		*		*	
Immunocompromised	*		*		*	
Liver disease	*		*		*	
HIV	*		*		*	
Barthel index ≤ 60 points	1.90 (0.47–7.72)	0.37	29.52 (4.51–193.16)	<0.001	32.27 (4.53–229.93)	0.001
Length of stay ≥ 30 days	3.27 (0.53–20.34)	0.20	*		9.11 (1.80–46.02)	0.008
ICU admission	5.23 (0.71–38.61)	0.11	*		*	
FINE-PSI on admission	0.48 (0.03–8.22)	0.61	1.45 (0.15–14.38)	0.751	0.85 (0.51–14.01)	>0.99
CURB-65 on admission	2.61 (1.06–6.41)	0.036	*		0.62 (0.19–2.04)	0.43
ARDS on admission	1.25 (0.52–3.03)	0.62	1.13 (0.43–2.97)	0.069	*	

Variables with clinical relevance and associations yielding a *p*-value of <0.10 in each univariate analysis are included. *: Variables with a *p*-value of ≥0.10 in each univariate analysis. ARDS: acute respiratory distress syndrome; CI: confidence interval; COPD: chronic obstructive pulmonary disease; ICU: intensive care unit; OR: odds ratio.

## Data Availability

On recruitment, a double-entry table was filled in linking the patient’s study number to their health card identification number. Subsequently, all data were entered into a hard-copy data collection notebook and finally into a password-protected electronic database in which only the study number appeared, ensuring anonymization.
